# Novel Peptide-Mediated Interactions Derived from High-Resolution 3-Dimensional Structures

**DOI:** 10.1371/journal.pcbi.1000789

**Published:** 2010-05-20

**Authors:** Amelie Stein, Patrick Aloy

**Affiliations:** 1 Institute for Research in Biomedicine, Joint IRB-BSC Program in Computational Biology, Barcelona, Spain; 2 Institució Catalana de Recerca i Estudis Avançats, Barcelona, Spain; University of Heidelberg, Germany

## Abstract

Many biological responses to intra- and extracellular stimuli are regulated through complex networks of transient protein interactions where a globular domain in one protein recognizes a linear peptide from another, creating a relatively small contact interface. These peptide stretches are often found in unstructured regions of proteins, and contain a consensus motif complementary to the interaction surface displayed by their binding partners. While most current methods for the *de novo* discovery of such motifs exploit their tendency to occur in disordered regions, our work here focuses on another observation: upon binding to their partner domain, motifs adopt a well-defined structure. Indeed, through the analysis of all peptide-mediated interactions of known high-resolution three-dimensional (3D) structure, we found that the structure of the peptide may be as characteristic as the consensus motif, and help identify target peptides even though they do not match the established patterns. Our analyses of the structural features of known motifs reveal that they tend to have a particular stretched and elongated structure, unlike most other peptides of the same length. Accordingly, we have implemented a strategy based on a Support Vector Machine that uses this features, along with other structure-encoded information about binding interfaces, to search the set of protein interactions of known 3D structure and to identify unnoticed peptide-mediated interactions among them. We have also derived consensus patterns for these interactions, whenever enough information was available, and compared our results with established linear motif patterns and their binding domains. Finally, to cross-validate our identification strategy, we scanned interactome networks from four model organisms with our newly derived patterns to see if any of them occurred more often than expected. Indeed, we found significant over-representations for 64 domain-motif interactions, 46 of which had not been described before, involving over 6,000 interactions in total for which we could suggest the molecular details determining the binding.

## Author Summary

Protein-protein interactions are paramount in any aspect of the cellular life. Some proteins form large macromolecular complexes that execute core functionalities of the cell, while others transmit information in signalling networks to co-ordinate these processes. The latter type, of more transient nature, often occurs through the recognition of a small linear sequence motif in one protein by a specialized globular domain in the other. These peptide stretches often contain a consensus pattern complementary to the interaction surface displayed by their binding partners, and adopt a well-defined structure upon binding. Information that is currently available only from high-resolution three-dimensional (3D) structures, and that can be as characteristic as the consensus motif itself. In this manuscript, we present a strategy to identify novel domain-motif interactions (DMIs) among the set of protein complexes of known 3D structures, which provides information on the consensus motif and binding domain and also allows ready identification of the key interacting residues. A detailed knowledge of the interface is critical to plan further functional studies and for the development of interfering elements, be it drug-like compounds or novel engineered binding proteins or peptides. The small interfaces typical for DMIs make them interesting candidates for all these applications.

## Introduction

Proteins are key players in all aspects of cellular life. They seldom act alone, but rather in combination with other molecules. Some proteins form parts of large macromolecular complexes that execute core functionalities of the cell, while others transmit information in signalling networks to co-ordinate these processes. To disentangle the complex network of protein interactions, both complex membership and binary interactions are currently being studied in large-scale experiments in several model organisms [1]–[6]. However, interaction discovery data mostly offers information on whether two proteins do or do not interact, but it cannot provide details on the mode of binding or the interaction interface. Atomic details of protein-protein interactions are only available in high-resolution 3-dimensional (3D) structures, which are collected in the Protein Data Bank (PDB) [7]. A detailed description of the atomic contacts involved in interaction interfaces often reveals the forces that hold two proteins together, and permits to extract conclusions on the potential disruptibility of the interface through, for instance, the action of specific drugs [8]. Recently, the combination of structural data and assignment of globular protein domains has allowed to distinguish between two main classes of protein-protein interfaces [9]: Domain-domain interfaces tend to be large and stable, while interfaces between a globular domain and a peptide stretch are usually smaller, sometimes with only a handful of key residues involved in the binding event [10], [11]. The latter type of interface allows for transient binding, making them ideal for signalling networks. The classification of interfaces into domain-domain or domain-peptide gives information on their size, strength, shape, and other features that may help us understand the interaction between the two proteins and how it reacts to competitors [12], their correct identification being thus critical. In both cases, high-resolution 3D structures provide crucial information on how proteins involved in these interactions recognize each other and achieve a high degree of specificity, which in the case of domain-peptide interactions also includes its context [11]. Furthermore, structures can also help identify key residues in binding pockets [13] or be used as the base of complex models for the prediction of domain-motif interactions (DMIs) [14]. It is thus clear that, the more high-resolution structural details we can compile on DMIs, the better we will understand their function and fast evolving profiles.

The peptides involved in DMIs are characterised by a consensus motif with specific conserved residues that are recognised by the binding domain. Some positions of such motifs are restricted to particular amino acids, while others may allow a set of similar residues, or even arbitrary ones. Consensus motifs are often given as regular expressions. For example, the Src-homology-3 (SH3) domain binds proline-rich peptides, and several variants of the PxxP (‘x’ or ‘.’ denote arbitrary positions) pattern have been observed, including [RKY]xxPxxP (class I; square brackets denote the set of possible residues for this position) and PxxPx[KR] (class II) [15]. Structurally, linear motifs are frequently found in disordered regions [16], thus exposed to potential binding partners and with the ability to adopt a variety of conformations [17], [18]. Most motifs assume a well-defined structure upon binding to their recognition domain, like the polyproline type II (PPII) helix adopted by SH3-binding peptides [15], the alpha helix, formed by Nuclear Receptor cofactor peptides [19] or the beta strand in peptides that interact through beta strand addition, as do PCNA- and PDZ-binding peptides [20]. Unstructured regions may adopt different conformations depending on the interaction partner, so that a given peptide could potentially bind more than one domain, each with the appropriate structure [21]. Given the small number of key residues, motifs can arise and vanish spontaneously with only a few mutations. Along with the modularity of their binding domains [18], [22], this allows a rapid evolution to explore novel regulatory interactions relatively easily [23]. In this way, a motif that mediates a particular function or interaction can arise convergently in otherwise unrelated proteins. Due to their transient nature, DMIs are difficult to identify in high-throughput interaction detection experiments [22]. In the last years, several methods have been developed for *de novo* discovery of motifs from sets of sequences assumed to share a feature that e.g. explains interaction with a common partner [24] or other biological factors like co-localisation or particular post-translational modifications [25]. These methods exploit the convergent evolution of linear motifs in looking for patterns that are over-represented among unrelated sequences in the query set. Homologous proteins or regions are removed before the computation of over-represented motifs, and domains or other well-structured regions are often masked because motifs tend to occur in unstructured regions [16]. Sometimes, though not always, the motif-binding domain can be identified in these *de novo* procedures for motif discovery [24]. A problem of these sequence-based motif discovery algorithms is the poor signal-to-noise ratio of many datasets [26], [27]. Focusing on the local environment of the motif, i.e., the *flanking regions* or *context*, increases the sensitivity of these methods [26] and may provide additional information in the search for functional interpretations of the novel motifs [27]. However, a potential caveat of methods relying on evolutionary conservation is that they might well miss some of the instances that have arisen very recently [26].

Despite the small size of their binding interfaces, domain-motif interactions are known to be highly specific *in vivo* [28], although they can also show some promiscuity, with similar affinities for native and non-native interaction partners, when tested in isolation [29]. Depending on the given binding domain, cell type and organism, the specificity may be encoded primarily in the motif sequence [13], [30], the flanking regions [11], or the network context [31]; probably these factors often work in concert. Traditionally, motif recognition patterns were split by one or two key residues (cf. [32]), but recent work has revealed that a much finer subdivision may be needed [33] or that, at least for some domains, there may not even be clear borders between the recognition profiles of different members of a domain family, but that recognition profiles cover the whole specificity space instead [28]. With their high specificity, regulatory function and small interface, DMIs make excellent candidates for drug targets [8], [34], [35], and information about high-resolution 3D structures of the interfaces may be crucial in this context [21], [36]. A recently published method by Petsalaki *et al.* [37] searches the surfaces of 3D stuctures for sites that may bind a given motif, based on physicochemical properties. If the binding domain or a set of possible binding domains for a motif are known, this tool could help identify the interface between peptide and domain. A successful prediction of the binding site on the domain would reveal much detail beyond what is given by sequence-based approaches, yet it would not provide the atomic contacts of the interaction. There are other computational tools, such as iSPOT [38], [39], that have been designed to predict peptide-binding specificities using also 3D structure information. In this case, however, one needs to know in advance that a given interaction is peptide-mediated and which are the exact residues participating in the interface.

While many current methods for the *de novo* discovery of motifs exploit the fact that they tend to occur in disordered regions, our work here focuses on another observation: upon binding to the domain, motifs adopt a well-defined structure (see also [40]). Indeed, the structure of the peptide may be as characteristic as the consensus motif, and help identify peptides even though they do not match the established consensus. An example is the linker peptide between the SH2 and kinase domains in the Src kinase [41], which adopts a PPII helix and is bound by the SH3 domain although it does not contain a PxxP motif. The interaction topology of the linker binding the SH3 domain is the same as that of intermolecular SH3-peptide pairs, so we consider them to belong to the same *interaction type* [42]. Domains may bind different kinds of peptides in different orientations [43]. During visual inspection of candidates for DMI based on experimentally confirmed motifs stored in the Eukaryotic Linear Motifs database (ELM) [32], we observed that there are several peptide-mediated interactions in structure which did not match the established consensus motifs of their corresponding interaction types and therefore could not be found by a procedure based on known patterns [11]. Furthermore, we noted that linear motifs have a particular stretched and elongated structure unlike most other peptides of the same length. We thus need to, somehow, use this information to identify more instances of DMIs in the databases, and derive the consensus binding patterns governing them. This would provide molecular details for many protein interactions discovered in high-throughput initiatives, and suggest the relevant mutagenesis experiments to tinker with them. In this manuscript we describe our studies of the structural features of known motifs, and use the results, along with other structure-encoded information about interactions, to scan through the set of protein complexes of known 3D structure in order to identify unnoticed peptide-mediated interactions among them. We compare our results with established linear motif patterns and their binding domains as described in ELM [11], and with other sources of structural descriptions of peptide-mediated interactions. Finally, we cross-validate our newly derived patterns on interactome data, and present a list of novel peptide-binding domains, along with their respective high-resolution 3D structures and consensus motifs.

## Results

### Structural parameters to capture linear motifs

To exploit structural features of peptides and domain-motif interactions, we first need to establish which parameters are suitable for the separation of known linear motifs from other, presumably non-functional peptides of the same length. We thus selected several structural parameters and applied them to the 631 DMIs of known 3D structure identified in our previous study [11], to test whether they could capture structural properties of functional linear motifs. To ensure that these parameters could partition peptides into true motifs and random cases, we created a control dataset based on the Structural Classification of Proteins (SCOP) [44]. For each SCOP fold, we chose one representative structure and generated all possible peptides of length 4–20 residues, which corresponds to the range of motif lengths in ELM. Although we cannot guarantee that SCOP folds do not contain true linear motifs, it is unlikely as they form well-defined tertiary structures, whereas motifs often occur in unstructured regions and outside domains. Therefore we assume that the SCOP control set constitutes a reasonable collection of negative instances for the identification of linear motifs.

**Figure 1:**
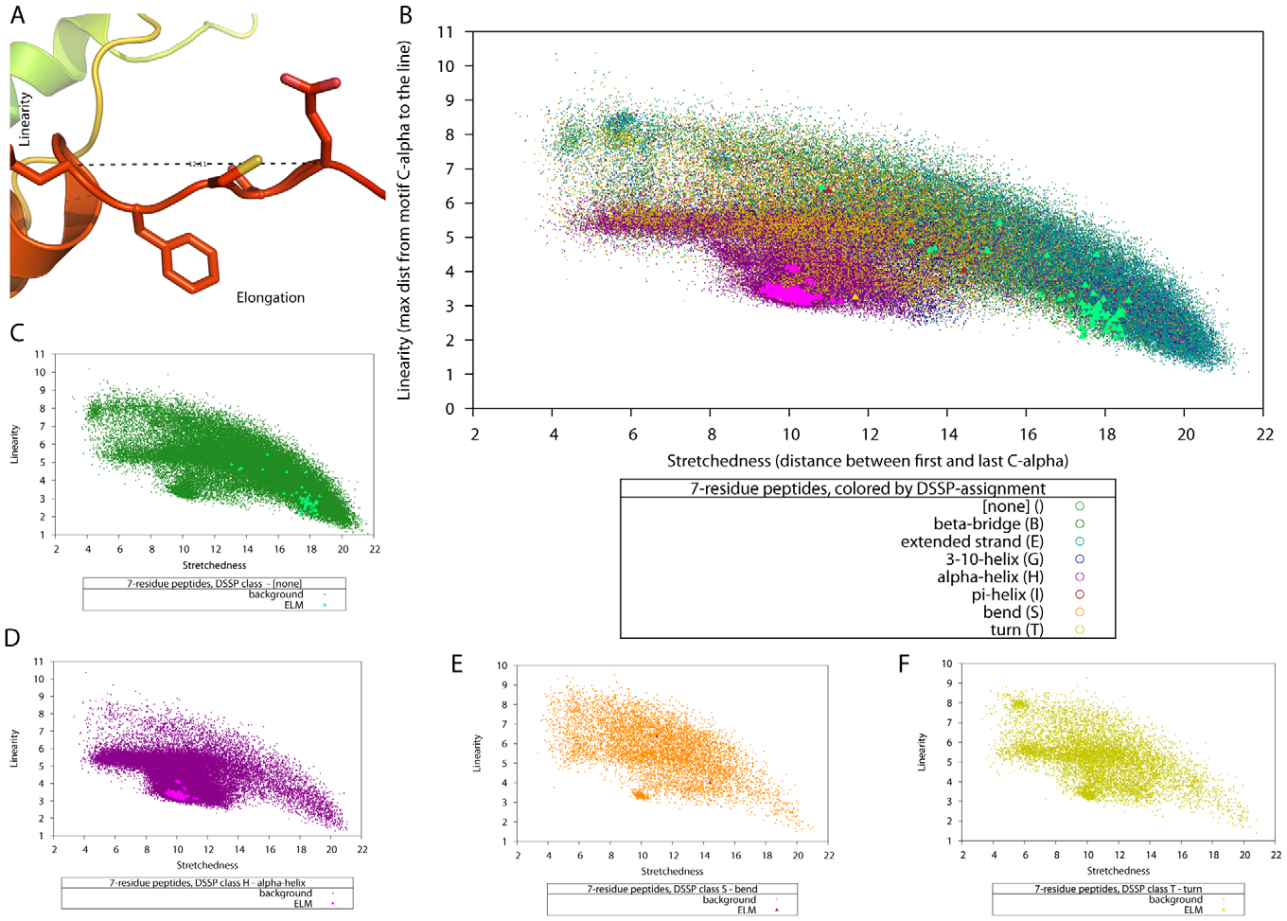
Linearity and elongation of linear motifs. (A) The Retinoblastoma-associated protein B domain (RB_B)-binding peptide shows the typical linear and elongated form found in 3D structures of many motifs (PDB ID 1gh6). The concepts of linearity (the maximum deviation of any 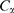 in the motif from the line through the first and last 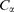) and elongation (the distance between the first and last 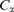 of a motif) are illustrated in this structure. (B) A slice of the data used for SVM training: linearity, elongation and secondary structure classification for 7-residue-peptides, with data from the SCOP background shown as dots and the data for known DMI shown as solid triangles, using one colour per DSSP classification. Panels (C) to (F) show the distribution of linearity∶elongation values for those secondary structure classifications for which we had known 7-residue-peptides (none, alpha-helix, bend, and turn). These data slices illustrate how known linear motifs fall into distinct regions of the parameter space.

**Figure 2:**
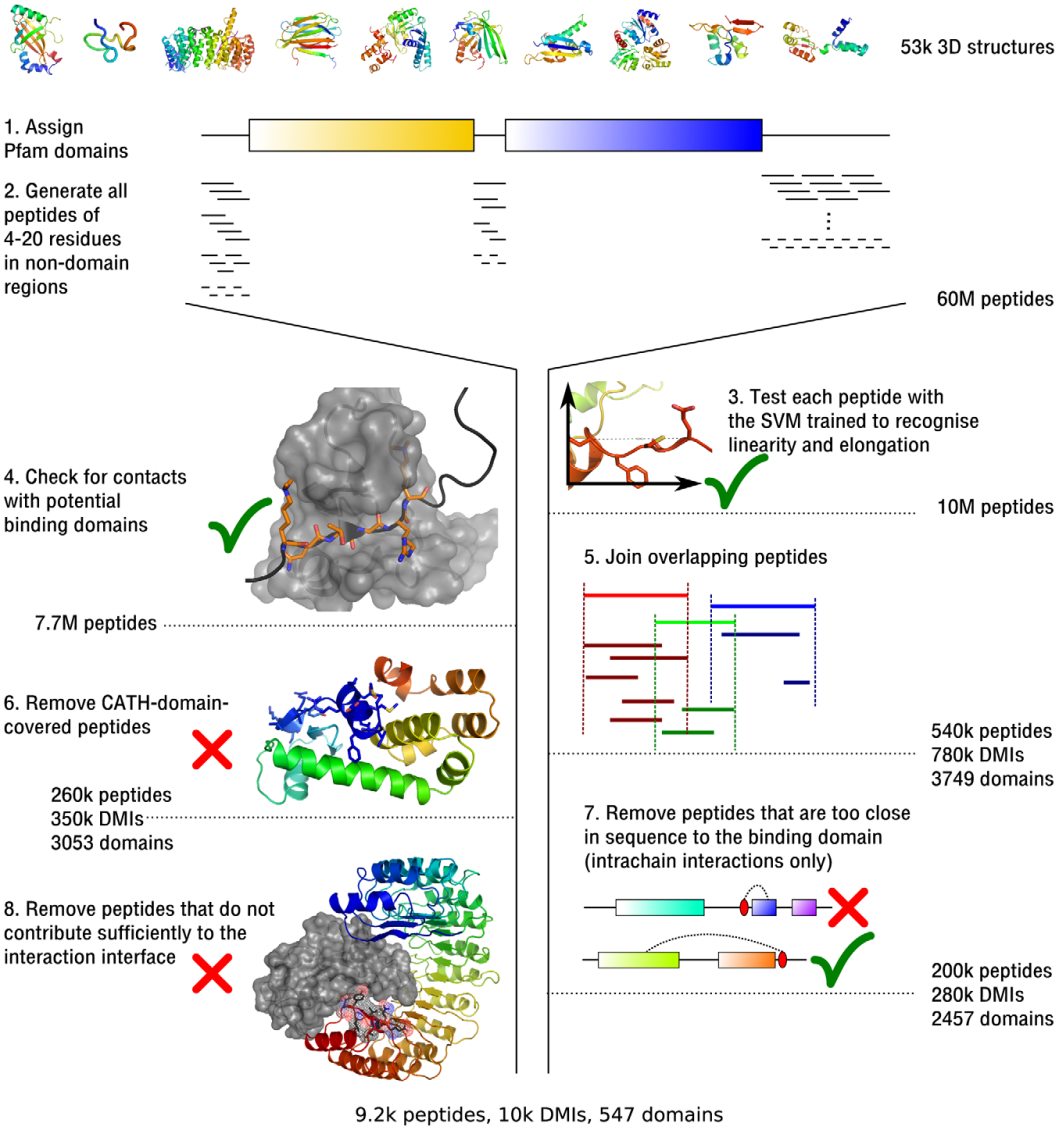
*Overview* of the generation and filtering of motif-like peptides. (Steps 1 and 2) We generated all possible peptides of 4–20 residues from regions of 3D structures that did not match Pfam domains. (3, 4) For peptides accepted by the SVM trained on linearity and elongation (cf. Figure 1) we computed whether there were sufficient contacts with domains in the same structure, which may be in the same or in another protein chain. (5) Peptides that are completely covered by other (longer) peptides are removed, so that the largest accepted peptide represents shorter candidates binding to the same region. (6) Peptides in intrachain interactions that are part of CATH domains are often artefacts of differences between structure- and sequence-based domain assignment and are therefore excluded. (7) Peptides in intrachain interactions that are sequentially directly next to the binding domain are often artefacts and thus removed, though in general peptides close to domains are allowed, as long as they have a sufficient sequential distance from their *binding* domain. (8) Exclude candidate DMI in which the interface is smaller than 150 Å, or in which the interface between domain and peptide is less than 50% of the total interface between the proteins.

The main structural parameters we developed for motifs are *linearity* and *elongation* (Fig. 1a). The *linearity* of a peptide is a marker of how “flat” it is, how much it deviates from a straight line through the first and last residue. The *elongation* indicates how long a peptide extends in space (Fig. 1a; for details see *Methods*). Together, linearity and elongation should capture our observations described above, namely that linear motifs are more flat and stretched than other peptides. Because flexibility and length of a peptide increase with the number of residues, it is important to only compare peptides with the same length in residues. We computed linearity and elongation for the known DMIs and the SCOP control set and found that, individually, neither of these parameters showed sufficient difference between the known cases and the SCOP control set, although there was a trend for known DMIs to be longer and more linear than other peptides of the same length in residues, confirming our observations (Figures S1a,b). We also assigned secondary structure to the peptides using DSSP [45] and observed that the distributions of values for linearity and elongation differed strongly between the classes of secondary structure. Helical structures were shorter and less linear, while beta sheets and unstructured regions were more linear and longer (Figures S1c,d). However, again the differences were not clear enough to use them to separate known DMIs from other peptides. Note that, as described above, some linear motifs act through beta strand addition, and yet others are known to form alpha helices, though most are found in unstructured regions. Since no single parameter was able to divide known DMIs from other peptides, we combined them to see if the trends described above would give a synergistic effect. For pure geometric considerations, a peptide that is flat should also be elongated in comparison to one that is helical or has bends and turns. Indeed, we found that known motifs fell into distinct regions of the space spanned by elongation and linearity, and are further subdivided by classes of secondary structure (Fig. 1b). Thus we concluded that these three factors can be used to separate structures similar to known motifs from other peptides of the same length in residues. Based on these findings, we set out to exploit structural data to find new instances of peptide-mediated interactions among the over 50,000 high-resolution 3D structures stored in the PDB (Fig. 2). However, the SVM will only recognise structural features of the peptide, but not consider any interactions to surrounding domains. Yet we cannot recognise DMIs based on the peptide alone, we need to take the interaction environment into account. We trained a support vector machine (SVM) with the data for linearity, elongation, secondary structure, accessibility and length in residues for all the known DMI peptides, and for a random set of 10,000 SCOP control set peptides (for details see *Methods*). Our first parameter that takes the environment of the peptide into account, accessibility, is required because peptides need to be accessible by other proteins in order to mediate interactions. Additional filters concerning the interaction environment will be described in the following paragraphs.

**Figure 3:**
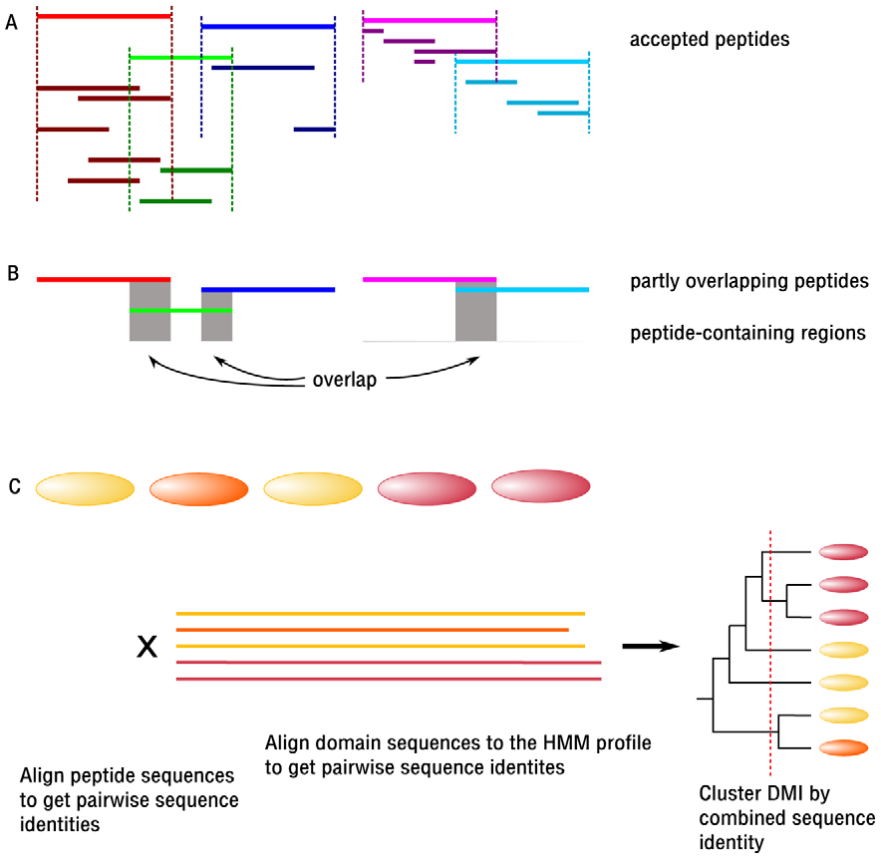
*Joining* of partially overlapping peptides for sequence-based clustering. (A) Partially overlapping peptides cannot be represented by either one, as both may contribute to an interface in ways not covered by the other. Yet to improve the quality of peptide alignments, and to ensure that motif matches in the overlapping regions (shown in gray) are only counted once for motif support, we need to create a construct that holds unique, non-overlapping regions of one or more peptides accepted by the SVM and having a sufficient interface with a domain. (B) Thus, for each continuous stretch of a protein that is covered by one or more peptides, we built a *peptide-containing region*. (C) These regions are then aligned to generate non-redundant sets of peptides binding to a given domain, and each motif match in a peptide-containing region only qualifies for motif support once. The 90% sequence clustering of the DMIs is computed from a combination of the sequence identities of peptide-containing regions and those of the binding domains.

We generated all possible peptides of lengths 4–20 residues from 52,903 3D structures, excluding regions covered by domains as assigned by Pfam HMMs [46] because motifs are rarely found in these. Note that this creates many overlapping peptides (cf. Fig. 2), which we generate in order to find the largest peptide that is accepted as *linear* and *elongated* enough. From the 60,123,359 candidates, only 10,596,512 (18%) peptides in 41,224 structures were accepted by the SVM. Next they were filtered for contacts to neighbouring domains in order to find putative domain-peptide interactions. We intended to identify all peptide-domain interactions, regardless of whether they appear within or between proteins. Therefore, for each candidate peptide that had been accepted by the SVM, we checked for contacts with domains in the vicinity, independent of whether they are part of the same protein or of another. We did not find enough contacts for 2,890,451 peptides (details see *Methods*), meaning that 7,706,061 peptides (73% of the accepted peptides; percentages in the motif discovery pipeline will always refer to the previous number of peptides or DMIs) in 40,199 structures remained. Next we removed some of the overlap that arises due to the way peptides are generated: If a short peptide has been accepted by SVM and domain contacts, and a longer peptide that includes the short one has been accepted as well, we only keep the long peptide (Fig. 3). Note that there may still be partially overlapping peptides in the set, in cases where none of the peptides covers the other completely. This overlap will be addressed later, as we cannot simply join peptides unless the resulting, encompassing peptide is also accepted by the SVM which tests for the typical structural features – linearity and elongation – of linear motifs. After the removal of completely overlapping peptides, we end up with 538,689 peptides (7%) in 40,199 structures, which are involved in 782,430 interactions, since one peptide may interact with several domains.

### Filtering candidate peptide-mediated interactions

At this step, we noticed that most of the DMI candidates corresponded to intrachain interactions (80%) and, upon visual inspection, many did not seem to be functional. These were often cases in which the domain and putative peptide, while in contact, had no extended binding surface, but rather protruding side chains touching each other, such that it was not clear whether this contact was biological or whether it might be an artifact that arose e.g. due to crystallization or buffer conditions. Other instances arose because of domain definitions that did not perfectly match the structure, in other words, when the Pfam domain assignments did not fully cover the structural units (folds), so that remaining elements (single strands or helices) were identified as binding peptides. Therefore we used domains in the protein structure classification CATH, which are defined on 3D structures [47], to filter out 426,464 (55%) intrachain peptide-mediated interaction candidates that were covered by these domains. Furthermore, many intrachain interactions were observed between a domain and a peptide close to the domain's boundaries. Accordingly, we removed 87,986 (25% of the remaining) interactions with a sequential domain-peptide distance below 10 residues. In addition, we filtered out contacts between proteins that are not listed in the Protein Quarternary Structure (PQS) database (2%), which contains presumed biological units of protein structures rather than the asymmetric units calculated in the structure determination process. The latter may bare signs of artefacts such as crystal packing. Also, during visual inspection we observed peptide candidates suggested to mediate interactions among multihomomers, which did not appear to be functional (visual inspection). As peptide-mediated interactions are usually heterologous, we removed cases in which the domain-containing protein and the peptide-containing protein form a homomer (1.4% of the candidate DMIs). Note that intrachain DMIs are heterologous as well, such as the SH3-peptide and SH2-phosphopeptide interacctions in the Src kinase described above [41], and structurally of very similar nature as their interchain equivalents [48].

Besides the key residues that form the consensus pattern, linear motifs are characterised by the fact that binding of the motif itself is sufficient to create a functional interaction (e.g., [49]). As we could not perform computationally expensive studies of binding energies for all candidate DMIs, we approximated the binding contribution of the peptide by comparing the domain-peptide interface with the full interface between the two partners. Specifically, we required the interface between domain and peptide to be at least 150Å^2^, which holds for over 90% of the known DMIs (Figure S2a), but may filter out putative interactions that are due to artefacts. 34,898 (14%) of the candidates had a smaller interface and were thus removed. In addition, to ensure that the peptide is a key player in the interactions detected in our procedure, we required the interface between peptide and domain to cover at least 50% of the total interface between the two proteins, which is true for 65% of the known DMIs (Figure S2b). The 50% threshold is intended to reflect our requirement for the domain-peptide-interface to have a major role in this interaction. Some peptide-mediated interactions are formed by multiple domains binding a peptide, e.g. the seven-blade beta-propellers formed by WD40 domains [50]. In those cases we only required all domain-peptide interfaces together to make up 50% of the full interface, and individual domains to contribute roughly equally (see *Methods*). Application of this filter removed 212,125 (95% of the remaining) putative DMIs, so that 10,739 candidates remained.

### Clustering of candidate interactions by sequence and topology

In order to identify unnoticed DMIs within the PDB, we needed to classify distinct domain-peptide interfaces and search for regular shared features among the peptides that explain why they bind the domain – a consensus motif. Thus, we needed to group the candidate interactions by topology to separate distinct interaction interfaces. Furthermore, due to the redundancy among entries in the PDB, we needed to create non-redundant sets of peptide-domain interactions. We only attempted to derive a consensus motif if sufficient non-redundant information was available. Note that we did not remove such redundancy in previous steps to capture as many variations of DMIs as possible.

**Figure 4:**
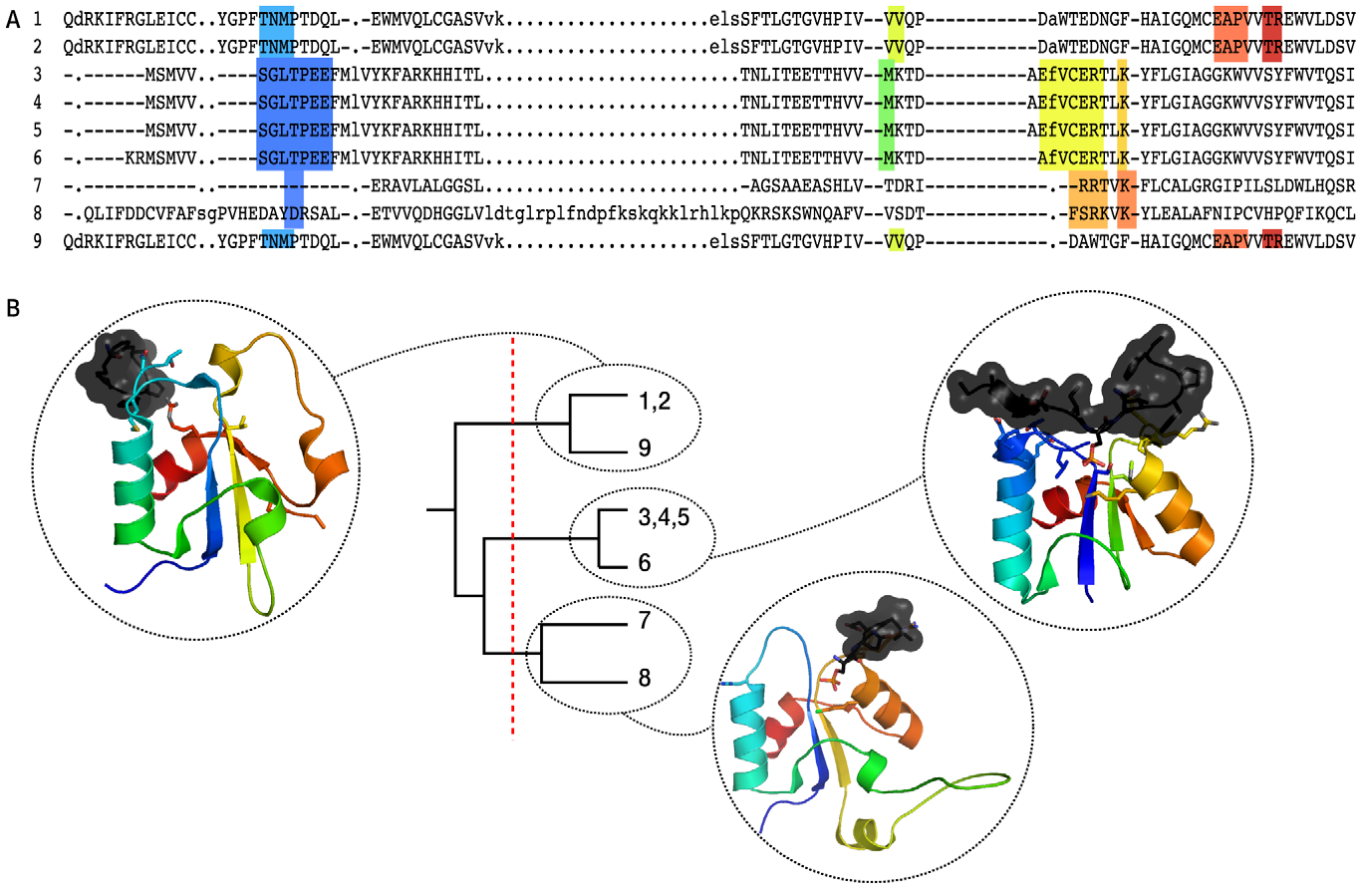
Topological clustering of peptide-mediated interactions. (A) Alignment of BRCA1 C Terminus (BRCT) sequences to the domain's HMM profile; interface residues are highlighted. The colour corresponds to the “rainbow” colouring scheme used for the domain visualisation in panel B. Lowercase letters refer to amino acids that do not match the domain's profile, - to positions in the profile that do not occur in the given sequence. (B) Clustering of the interaction topologies, based on shared interface residues. Domains with the same or highly similar topologies are grouped together. In the structural representation, all three BRCT domains have the same orientation. Note that the BRCT domain usually forms dimers that bind the peptide, using the interfaces from clusters (3,4,5,6) and (1,2,9), respectively (cf. Figure 5).

The topological clustering procedure we developed focuses on the residues forming the interface. We computed the fraction of shared peptide-binding residues between each pair of domains from a family, mapping corresponding residues via alignment to Pfam's HMM profile for that family (see Fig. 4 and *Methods*). Next we clustered the interfaces based on the shared peptide-binding residues to separate all interactions for this domain into distinct interfaces. Our method is similar to that by Teyra *et al.*, [51] but relies on multiple instead of pairwise alignment of the domains. In total, we found 822 topological clusters or interaction types, including 547 domains. The largest clusters contain over 700 DMI instances. Domains with many different peptide-binding topologies include protein kinases (8), trypsin (14), Pyridine nucleotide-disulphide oxidoreductase (Pyr_redox_2, 15 topologies), and the immunoglobulin V-set domain (18). However, note that these potential ligands have not been examined for significant motifs yet, so they do not necessarily represent functional DMIs. The sequence-based clustering, which is independent of the topological clustering, serves the creation of a sequentially non-redundant set of peptides bound by a given domain for the derivation of consensus patterns. It is used to create groups of “sufficiently different” peptides per domain, to establish which peptides are different enough to qualify for *motif support*, for which non-redundant data is required. Ideally, a set of unrelated sequences would allow over-represented motifs to be detected easily, as similarities cannot be due to larger conserved regions. To this end, first we need to address the issue of partially overlapping peptides that arises from the way peptides are generated (Fig. 2). A sequence-based clustering procedure working on the pure peptides, which are at most 20 residues long, could not detect small overlaps. If such a small overlap were to contain a motif, it would then be supported (counted) twice. To avoid these duplicated counts, we first joined peptide-containing stretches such that each protein section that was continuously covered by peptides (i.e. without gaps) was combined into a single region (see Fig. 3). Then these non-overlapping regions were aligned, and their pairwise sequence identity was computed. Clustering by sequence identity was based on combined sequence identity scores for both domain and peptide (see *Methods* for further details). In total, we found 2,490 clusters of 90% sequence identity for the 547 domains, with the largest clusters having over 200 entries. The immunoglobulin V-set domain shows the greatest sequential diversity in its ligands (220 clusters), followed by trypsin (108) and Major Histocompatibility Complex I (105). We are fully aware that 90% sequence identity is a more stringent threshold than what is normally used when creating sets of unrelated proteins. Our reasons to apply such a strict criterion are twofold: we are handling relatively short peptides, on which alignment does not always work reliably, and by selecting a lower threshold, such as 25% or 50%, we would risk getting too different peptides within the same cluster. In addition, we also need to identify motifs even among sets of proteins that are relatively similar (i.e. motifs occurring in the same protein family). Thus the sets of proteins in our clustering should be considered as *non-redundant* rather than *unrelated*. Nevertheless, we explored the possibility of relaxing the sequence similarity threshold to 50% in the clustering procedure (data not shown), and found that, since the clusters are broader and cover more instances, the number of interaction types with sufficient non-redundant information to derive significant patterns (see subsequent paragraph) dropped from 224 to 96.

### Deriving consensus motifs

For each topological cluster with at least 3 non-redundant sequences, we attempted to derive a consensus motif using SLiMFinder [25]. SLiMFinder identifies convergently evolved linear motifs in a set of sequences based on their occurrence in unrelated sequences, and computes a probability of their significance. It often suggests more than one possible motif, ranked by their probability of arising by chance. The program requires information about the evolutionary relationship of the motif-containing sequences, in particular which of them are so closely related that they should be considered a single case of support for a candidate motif when it is examined for whether there are enough unrelated proteins matching it. We provided the 90% sequence identity clusters for this purpose, so that only non-redundant cases are counted for motif support. Among our 822 topological clusters or interaction types, only 224 contained at least 3 non-redundant sequences. These covered 157 domains, with up to 13 clusters per domain. In addition to the sequences and their evolutionary grouping, we included information on modified residues in the peptides, because some recognition domains, like SH2 or 14-3-3, specifically bind peptides that have been post-translationally modified, e.g., by phosphorylation or methylation [18], [52], [53]. The domain's binding groove recognises the residue with the post-translational modification, so it should be a crucial element in a consensus motif. We searched all accepted peptides for modified residues, and if a particular residue was modified in more than half of the peptides in a cluster, it was required for the motif. Furthermore, we checked for helical peptides, which are another special case for pattern derivation, as helical structures create a regular pattern of residues pointing towards the domain vs. residues pointing away from it. If over 50% of the peptides in a cluster were helical according to DSSP, we enabled the helical pattern derivation feature of SLiMFinder, which takes this particular spacing into account.

For 152 of the interaction types, covering 111 domains, we found at least one significant motif, and for 96 of these, significant motifs were found for all topological clusters (interaction types). The 46 remaining domains did not yield any significant motif, although 3 or more non-redundant sequences were available for pattern derivation. As a curiosity, 36 of the interaction types with significant motifs (31 domains) involved helical peptides, and 20 (18 domains) required at least one modified residue in the pattern. In total, 5,316 interactions in 3D structures are covered by these 152 interaction types for which we could derive a significant motif, including 4,202 inter- and 1,114 intra-chain interactions, respectively. However, 16 clusters among 15 domains contained only intrachain interactions that, upon visual inspection, did not seem to be functional peptide-mediated interactions and were thus excluded. In addition, 8 putative DMI interaction types are always found between proteins that also have a domain-domain interface (DDI), which presumably is more reliable. Our assumption here is that if the DMI is functional, it should also occur independent of a DDI. Hence we modified our method not to accept clusters unless there are interchain instances, and DMI that appear without a DDI in the same protein pair. It is interesting to note that only 18 of the 94 domains for which we find significant patterns in the full dataset are described in ELM. It should be noted though that ELM does not always provide Pfam domain names and thus the overlap could be slightly larger and we are just not able to detect it.

### Benchmark of the DMIs identification accuracy

To assess the performance of our method in detecting peptide-mediated interactions and discarding non-functional peptides or interfaces in the PDB, we created a benchmark set of 631 known DMIs [11] and 631 random peptides from the SCOP dataset that do interact with a domain in a different protein (i.e. we only kept interchain training data) and are not fully covered by a domain. To ensure that DMIs are recognised by features beyond similarities among homologous domains and their binding peptides in the training set, we performed the benchmark in a *leave-one-domain-out* fashion, i.e., we removed all peptides binding to a given domain from the training set, and tested the recovery of the corresponding interactions and the detection of its consensus motif using the resulting SVM. For example, in one instance we left out all SH2-binding peptides (the *test set*), then re-ran the full motif discovery pipeline as described above and finally tested how many of them were rediscovered by the SVM trained on the remaining, non-SH2-binding peptides (the *training set*). If a peptide overlapping in at least 3 positions with a test set peptide was accepted and a significant pattern for its domain and topological cluster could be derived using SLiMFinder, the case was classified as “positive”, otherwise (peptide not accepted or non-significant pattern) as “negative”. For known DMIs, we also tested whether the known consensus motif given in ELM scored significantly using SLiMSearch [54], which works similarly to SLiMFinder but allows checking the significance of a predefined motif on a given set of sequences.

After applying the described procedure, we could automatically rediscover 423 of the 631 known DMIs interaction types, which correspond to a sensitivity of 68%. In terms of domains, we correctly recovered cases for 20 out of the 30 domains, i.e., the domain-based sensitivity (67%), very similar to that based on individual cases. Our method did not accept any of the negative cases from the benchmark, indicating that it is highly specific. Analyses on the 208 known DMIs that we could not recover showed that almost half of them (42%) can be explained by the fact that they are covered by domains and thus never considered as peptide candidates by our method, while manually curated sets like ELM and our previous study [11] did not apply such a filter. Other reasons for non-rediscovered positive cases included insignificant patterns (21%) or no pattern determined because of a lack of data (12%), too few contacts between domain and peptide (8%), and insufficient surface contribution of the domain-peptide interface (7%), among others. There are three domains for which the benchmark returns positive results, but no significant pattern is found when applying our method to the full PDB, which may be due to differences in the data set size in significance computation. Note that cases with too few non-redundant sequences were ignored (i.e., they are not counted as false negatives) for both negative and positive test cases. As an additional independent benchmark, we tested how many of the peptide-mediated interactions from the benchmark set by Petsalaki *et al.* [37] could be identified with our approach. In total, we recovered 298 of the 405 DMIs in their set (74%), which is slightly above the 240 cases (59%) that they correctly predicted from their benchmark. Analysis of the results showed that half of the instances that we missed are due to a low surface contribution, while the other half was covered by domains or not accepted by our SVM.

### Cross-validation with interactome networks

**Figure 5:**
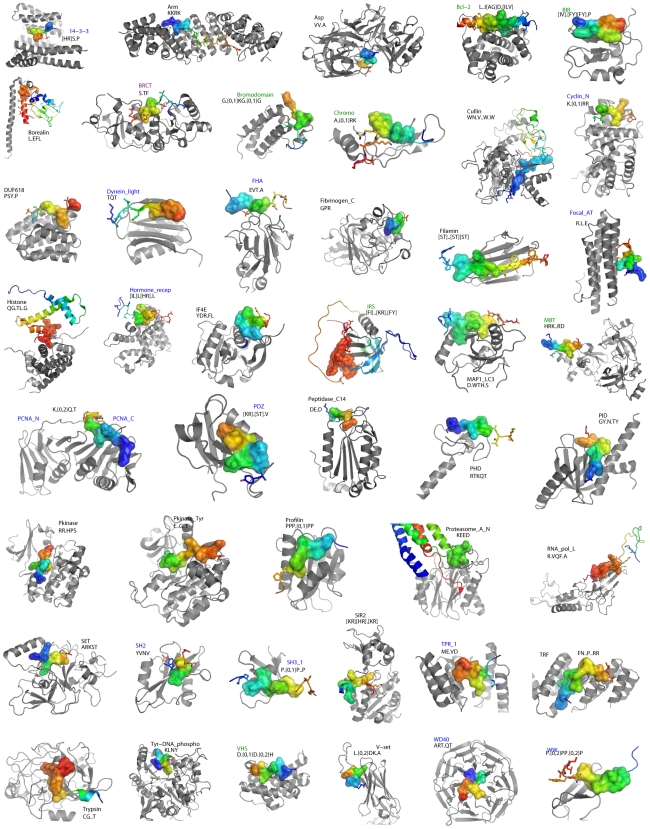
*DMIs* significantly enriched in the interactomes. Significantly enriched motifs were found for 46 distinct domains (shown in gray; PCNA_N and PCNA_C are shown in the same structure). Binding peptides are given in a rainbow colour scheme, with the SVM-accepted part in sticks representation and the consensus motif in surface representation. In most cases, differences between the interaction types for a given domain are subtle, thus only one is shown in this representative figure. However, for domains that form repeats to bind peptides (Arm, BRCT (cf. Fig. 4 and main text), TPR_1, TRF, WD40), we have visualized all domains required to bind one peptide; these usually employ different interaction types. Blue domain names indicate those that were described in the ELM training dataset [11], violet names mark additions to ELM since 2007 [32], which were not in our training set, and green names indicate DMIs that are described on the Pawson lab web site [60] but not in ELM.

To assess the validity of each motif derived by SLiMFinder and confirm that it could indeed occur in different protein interactions, we checked whether it was over-represented in proteins known to interact with a partner that contains the respective binding domain (see *Methods*). For example, although motifs tend to be degenerated, 50 of the 593 (8.4%) human proteins that interact with other proteins containing 14-3-3 domains match the ELM pattern *R[SFYW].S.P*, but the number is reduced to only 206 out of the 7215 (2.8%) of the proteins not interacting with any 14-3-3-containing protein. This corresponds to an enrichment factor of 2.57, which is statistically significant (p-value 2.827e-10, one-sided Fisher's exact test). We used interactome data for selected model organisms with relatively good coverage (yeast, worm, fly, human). We only tested motifs binding to domains of which a structure was solved in this species, to make sure that there is a functional occurrence of it in the species in question, so this validation procedure was limited to 64 domains among the 111 for which we suggest binding motifs. To avoid false positive hits, we required pattern matches be outside of globular domains, as do many sequence-based tools for motif detection [24], [25]. In addition, we only counted those motif-containing proteins as support for the DMIs if the interaction cannot be explained by a domain-domain interaction between that protein pair [10]. For each domain, we tested all patterns derived by SLiMFinder with the given parameters (see *Methods*) that is found in one of the selected interactomes. We then computed whether proteins interacting with a partner containing the recognition domain are enriched in hits for the derived motif (one-sided Fisher's exact test, p-value threshold 0.025). We found significant enrichments for 64/90 of the interaction types and 46/64 of the domains, with 1 to 6 patterns per interaction type enriched. Fig. 5 shows structures for each interaction type found to be enriched in the interactome cross-validation along with its most significantly enriched motif, which is not necessarily the top-ranked by SLiMFinder. Across the interactomes of the four model species considered in this cross-validation, our DMIs described here could offer molecular bases for over 6,000 interactions: 5199 in human, 160 in fly, 19 in worm and 941 interactions in yeast. Applying the statistical test to the 44 known ELM motifs for which we found a 3D structure [11] reveals significant enrichment for 72% of the DMIs and 74% of the domains, which is slightly higher than that observed for the patterns derived in this work. Looking at all 66 ELMs we considered for our previous study (i.e., also including ELM patterns for which we did not find occurrences in 3D structure), we find significant enrichments for 55% of the DMI and 59% of the binding domains. This decrease might suggest that motifs with known 3D structures are somehow better defined. Table 1 shows the list of DMIs for which we found a significant enrichment in the interactome networks, together with the best-ranked pattern according to SLiMFinder and the most significantly enriched. The complete list of patterns is provided in Table S1.

**Table 1:** DMIs significantly enriched in the interactome cross-validation.

Domain	TC	top-ranked pattern	Enrichment	p-value	most enriched pattern	Enrichment	p-value
**14-3-3**	0	[HR]S.P	1.31	0	SHSY	3.63	0.001
**14-3-3**	2	LDL	1.19	0.007	LD.{0,1}L	1.22	0
	R[SFYW].S.P (LIG_14-3-3_1), R.[SYFWTQAD].[ST].[PLM] (LIG_14-3-3_2), [RHK][STALV].[ST].[PESRDIF] (LIG_14-3-3_3)						
**Arm**	0	KK[KR]K	1.45	0.043	KKRK	2.25	0.001
**Arm**	1	KKRKV	2.13	0.383	K[KR].K[LV][DE]	3.04	0.001
**Arm**	2	KK[KR]K	1.45	0.043	KKRK	2.25	0.001
**Asp**	2	HPFH	0	1	VV.A	2.58	0
**Bcl-2**	2	QL..I[AG]D	0	1	R.I[AG]D.[LV]	13.56	0.009
**BIR**	0	AVP[FI]	8.87	0.107	[IV].[FY][FY].P	25.16	0
**Borealin**	0	L.EFL	33.95	0	L.EFL	33.95	0
**BRCT**	0	S..FP.A	1.2	0.501	D..QVF.F	23.55	0.002
**BRCT**	1	SPTF	2.14	0.116	S.TF	1.56	0.01
**Bromodomain**	2	[GS].GG	1.36	0	GKG.{0,1}GK	8.8	0
**Chromo**	2	ARK[ST]	2.27	0.07	T.{0,2}ARKS	9.62	0.003
**Cullin**	0	WN.V..W.W	86.76	0	W..V..W..DI	86.76	0.012
**Cyclin_N**	0	K.{0,1}RRL	1.13	0.458	KR.L..E	3.01	0.003
	[RK].L.{0,1}[FYLIVMP] (LIG_CYCLIN_1)						
**DUF618**	0	PSYSP	0	1	PSY.P	56.58	0.001
**Dynein_light**	0	K.TQT	6.2	0.15	TQT	4.11	0
	[KR].TQT (LIG_Dynein_DLC8_1)						
**FHA**	0	EVTE.D	25.86	0.039	LE.TE	5.32	0
	T..[ILA] (LIG_FHA_1)						
**Fibrinogen_C**	0	HRP	2.37	0.085	GPR	2.25	0.014
**Filamin**	0	[KR]S[AS]	1.2	0.066	[ST]..[ST][ST]	1.17	0.021
**Focal_AT**	0	R.L.E	1.56	0.022	LSE	1.81	0.002
	[LV][DE].[LM][LM]..L (LIG_PXL)						
**Histone**	0	Q.RT.Y.F	0	1	QG.TL.G	40.89	0.001
**Hormone_recep**	2	[IL]L[HR].LL	0.7	0.806	[IL]L[HR].L	1.36	0.02
	L[ ^P]{2}[HI]I[ ^P]{2}[IAV][IL] (LIG_CORNRBOX), [ ^P](L)[ ^P][ ^P](L)(L)[ ^P] (LIG_NRBOX)						
**IF4E**	0	YDR.FL	156.16	0	YDR.FL	156.16	0
**IRS**	0	[FI]..[KR].[FY]	1.48	0.003	[FI]..[KR].[FY]	1.48	0.003
**MAP1_LC3**	0	D.WTH.S	108.44	0.009	D..THLS	108.44	0.009
**MBT**	0	HRK..RD	56.58	0.018	RKV.RD	339.48	0.003
**PCNA_C**	0	K.{0,2}QATL	34.22	0.029	K.{0,2}Q.T	1.6	0.021
**PCNA_N**	1	K.{0,2}QATL	34.22	0.029	K.{0,2}Q.T	1.6	0.021
	(^{0,3}|Q).[ ^FHWY][ILM][ ^P][ ^FHILVWYP][DHFM][FMY].. (LIG_PCNA)						
**PDZ**	0	RETQV	0	1	R.ET.V	2.89	0
	.[ST].[VIL]$ (LIG_PDZ_1), .[VYF].[VIL]$ (LIG_PDZ_2), .[DE].[IVL] (LIG_PDZ_3)						
**Peptidase_C14**	1	D.SD	1.36	0.028	DE.D	2.18	0
**Peptidase_C14**	4	DE.D	2.18	0	DEVD	4.1	0
**PHD**	0	RTKQT	11.52	0.011	A.TK..AR	17.29	0
**PID**	2	Y.NP.YK	0	1	GY.N.TY	68.15	0.015
**Pkinase**	3	RRRHP	1.02	0.675	RR.HPS	4.08	0.015
**Pkinase**	6	T.NL	1.14	0.024	T.NL	1.14	0.024
**Pkinase_Tyr**	2	EIF..FE	0	1	E.FG..E	2.43	0.021
**Profilin**	0	PPP.{0,1}PP	4.48	0	PPP..P.P	8.6	0
**Proteasome_A_N**	0	K.EDN.G	0	1	KEE..L	3.1	0.007
**RNA_pol_L**	0	T.R..QF..R	32.47	0.031	R.VQF.A	21.64	0.003
**SET**	0	AR.{0,1}K.T	2.28	0.068	ARKST	20.55	0
**SH2**	0	YVNV	3.85	0	HIYDE	8.18	0.015
**SH2**	1	S.TIYA	4.09	0.229	IY.QVQ	8.18	0.015
	Y.N. (LIG_SH2_GRB2), Y[IV].[VILP] (LIG_SH2_PTP2), Y[QDEVAIL][DENPYHI][IPVGAHS] (LIG_SH2_SRC), Y..Q (LIG_SH2_STAT3), Y[VLTFIC].. (LIG_SH2_STAT5)						
**SH3_1**	0	P.{0,1}P.{0,2}P.{0,2}P	2.2	0	P.PV.{0,1}PP	9.22	0.019
**SH3_1**	1	P.{0,1}P..P	1.8	0	P.{0,1}PP.{1,2}P	3	0
**SH3_1**	2	DR.TKP	1.72	0.468	DR.T	1.44	0
	[RKY]..P..P (LIG_SH3_1), P..P.[KR] (LIG_SH3_2), …[PV]..P (LIG_SH3_3)						
**SIR2**	0	HKKLM	0	1	[KR][HR].[KR]	1.46	0.008
**TPR_1**	0	EEVD	2.01	0.027	ME.VD	4.02	0.007
	EEVD$ (LIG_TPR)						
**TRF**	0	[FY].L.P[LV]	2.67	0.172	FN.A..GR	244	0.004
**Trypsin**	1	PG.Y	1.87	0.001	PG.Y	1.87	0.001
**Trypsin**	3	CGK	0.66	0.925	CG..T	1.86	0.015
**Trypsin**	5	PAIQP	0	1	P.IQ	1.59	0.018
**Trypsin**	7	CT..IPP	0	1	CT..I.P	8	0.024
**Trypsin**	11	CG.[KR]	1.24	0.189	CG..T	1.86	0.015
**Trypsin**	12	[FY]E.IP.E	0	1	DF..IP.{0,1}E	14.39	0.007
**Tyr-DNA_phospho**	0	KLNY	177.45	0.006	KLNY	177.45	0.006
**V-set**	3	Q.DPAF	15.93	0.062	K..[HK].G	1.38	0.008
**V-set**	10	E.DKW	5.31	0.053	A.FRHD	15.93	0.006
**V-set**	11	WF..T..LW	0	1	QE..D..RE	10.62	0.014
**V-set**	15	D.PDY.S	0	1	P.Y.S	1.79	0.001
**V-set**	16	E.DKW	5.31	0.053	L.FGYP	31.87	0.001
**VHS**	0	D..LL	1.12	0.314	DL..I	1.72	0.012
**WD40**	0	RTKQT	6.71	0.006	TKQTA	8.39	0.003
	F.[IV][ ^WFY][ ^WFY][IL][ILM] (LIG_EH1)						
**WW**	0	P.{0,2}PP.{0,2}P	1.64	0	PPPY	6.24	0
PP.Y (LIG_WW_1), PPLP (LIG_WW_2), …[ST]P. (LIG_WW_4)							

List of domains and motif patterns determined in this study and found to be significantly enriched in the interactome cross-validation. The top-ranked SLIMFinder motif and the pattern with the highest significant enrichment are shown for each topological cluster (TC), along with the respective enrichment and p-values. If available, ELM ligands of the domain are given below the patterns derived in this work. A table with all DMI, including those not enriched in the interactome cross-validation as well as the 3 top-ranked patterns for all candidate DMIs is given in Table S1.

### A look at specific examples

Although it is the general trends that conform the main message of this work, it is always illustrative to look at some specific examples to understand the nature of our results. Among the novel DMIs identified by our approach there are two that, in the meantime, have been included in ELM, but were not listed when our training set was built. These can be considered “blind tests”, since neither the SVM nor other filtering parameters were selected using the information in these domains and peptides. One case is the BRCA1 C Terminus (BRCT) domain, which forms dimers that bind a phosphopeptide [55]. The different orientations of the domain with respect to the peptide are recognised in our procedure, and two topological clusters are generated (see also Figure 4). The best-ranked pattern is *S..FP*; where the S is always found phosphorylated. The ELM annotation also describes a phosphorylated S, along with two similar patterns (.*S..F* and .*S..F.K*).

The second DMI from our results that has been added to ELM is cytoskeleton-associated proteins domain (CAP_GLY), which is involved in the regulation of microtubules [56]. It recognizes short D/E-containing peptides; the consensus motif that we derived is *DE.F* (or *D.{0,1}E.F*) while the ELM pattern is a much longer one: *[ED].{0,2}[ED].{0,2}[EDQ].{0,1}[YF]$*. ELM contains the additional information that the peptide is always C-terminal, indicated by the $ symbol. In our approach we do not try to establish whether a pattern occurs at one of the termini, because peptides in 3D structures are often truncated, so that what appears to be a terminus won't necessarily be one *in vivo*. In this case, however, neither the ELM CAP_GLY pattern nor the one we derived is significantly enriched in proteins interacting with those containing the domain.

A very interesting example is that of the Bcl-2 protein family, which is crucial in the regulation of apoptosis and has both pro-apoptotic and pro-survival members [57], [58]. Many of these are multidomain proteins that contain four conserved Bcl-2 homology (BH) domains. In addition, some members of the extended family only contain one of the BH domains, BH3, which forms a helical peptide that can be bound by multidomain Bcl-2 proteins [59]. As survival despite pro-apoptotic signals is a problem in many cancer cells, this family comprises several interesting drug target candidates. Indeed, a number of small molecule agonists and antagonists have recently been developed and are currently in various stages of clinical trials [59], some of which have been developed based on 3D structures of Bcl-2 and its binding peptide (the BH3 domain). The family is also listed as a peptide-binding domain on the Pawson lab web site [60], named BH1-BH2-BH3-BH4, but a consensus motif for the peptides is not given. The top-ranked significant motif we identified is *L..I[AG]D.[ILV]*, with the large hydrophobic residues pointing into the binding groove. Two other, very similar motifs scored significantly (*LR.I.D.[LV]* and *R.I[AG]D.[LV]*); both also contain the large hydrophobic residues in the appropriate spacing pattern. Structurally, the peptide is always helical, so one might consider replacing the arbitrary positions (.) by anything but proline [ ^P], because of the helix-breaking properties of this aminoacid. This restriction is also found in other motifs, such as ligands of hormone receptors (Hormone_recep), another all-alpha protein that binds small helical peptide ligands [19].

Finally, in contrast to the three examples above, we could not identify a significant motif for Clp protease (CLP_protease), although sufficient non-redundant sequences were available. Given that Clp proteases degrade peptides with little sequence specificity [61], the fact that our approach could not identify a defined consensus motif should be considered positive for our method.

## Discussion

The identification and correct classification of domain-motif interactions is a key issue to understand the biophysical principles governing interactome networks, such as the relationship between protein-binding domains and the consensus motifs they recognize. Accordingly, we have presented a method to indentify unnoticed domain-motif interactions (DMIs) among high-resolution 3D structures, which not only provides information on the consensus motif and binding domain, but also allows ready identification of the key residues on both the motif and the domain side. Applying this methodology to all currently available 3D structures has revealed 152 DMIs, 127 of which have not been described previously. Moreover, 64 of the motifs have been found to be significantly enriched in proteins interacting with those containing the respective binding domain. In a *leave-one-domain-out* benchmark on the 3D structures of known ELMs [11], our method could rediscover and compute consensus motifs for 2/3 of the known cases. In addition, it is very precise as none of the random motif-domain pairs we tested as negative control cases were accepted. As far as we know, no other method for *de novo* motif discovery can provide such details for a novel DMI. Indeed, few other methods exploit the information encoded in 3D structures, although the importance of the 3D structure of motifs and their flanking regions for functional analysis has recently been highlighted [27]. The information that a peptide adopts a particular structure in interaction, even though it may be unstructured on its own, is currently only available from high-resolution 3D structures. While sequence-based methods for motif discovery have the advantage that they are applicable to larger datasets, they cannot necessarily reveal the binding domain of a suggested motif [24], or the atomic details of the interface. Knowing the interface of an interaction, however, is critical for functional studies [12] as well as the development of interfering elements, be it drug-like compounds [21], [36], novel binding proteins [62] or engineered peptides in synthetic circuits or networks [63], [64]. The transient interactions and small interfaces in DMIs make them interesting candidates for both applications. With previous large-scale methods, gaining this knowledge in one step was not possible. Only once a pattern and its binding domain have been identified, the recently published method by Petsalaki *et al.* [37] can be used to search structures of this domain for surface patches that are complementary to the pattern, although it has some difficulties with helical and beta-strand-forming peptides. In addition, without further information, that method cannot show which residues in the domain interact with the key residues in the motif, and would thus benefit form a combined strategy with the approach presented here.

A different issue with the sequence-based quest for motifs is that the “shared feature” may be too loosely defined. For example, there are many kinases that phosphorylate [ST] or Y residues, yet it would not be possible to derive a meaningful motif from the set of phosphorylated sequences alone. Our method instead focuses on the atomic interface between peptide and domain, which includes a concise definition of the environment in which the motif is bound. This, in turn, ensures that all peptides in one group for pattern derivation use the same interface, which is a rather strict definition of “shared feature”. An additional advantage is the direct possibility of visual inspection of the suggested DMIs. The main bottleneck is the availability of domain-peptide interactions in 3D structures in large enough numbers and with sufficient diversity to allow for the derivation of a consensus motif. However, if this information is available, the results are highly specific and contain a level of detail that cannot be provided by other techniques. Furthermore, our method successfully detected helical peptides or those acting through beta-sheet addition, which present difficult cases for the other 3D-structure-based method for DMI interface detection [37]. Yet, while our method successfully identifies DMIs in helical peptides, we do not fully exploit the information provided in those structures – for the peptide to be helical, it should not contain proline residues [65]. However, even though no prolines occur in the sequences, SLiMFinder cannot determine that this residue is “forbidden” because the amount of information encoded in the sequences used for training is much too small for such conclusions. Only studies on all possible sequence variations, such as phage display data, could allow the derivation of forbidden residues in certain positions. However, one might consider manually modifying the patterns of helical peptides to reflect this, by replacing all arbitrary positions (.) by [ ^P], as it has been done e.g. for the hormone receptor ligands in ELM (pattern [ ^P](L)[ ^P][ ^P](L)(L)[ ^P], parentheses indicate that the leucines are key residues).

We chose strict thresholds on contacts and interface size to limit the occurrence of false positives, which are often problematic when dealing with so few key residues as in motif-mediated interactions. While our high precision shows the advantages of those strict thresholds, we do miss some true motifs as described in ELM, in particular due to the exclusion of regions assigned to Pfam domains, which is responsible for almost half of the true motifs we do not recover. Yet the inclusion of these regions would disproportionately increase the computation time as well as the risk of false positives, since it has been shown that motifs usually occur outside of domains [16]. Nevertheless, it may be possible to create a fine-tuned version of our method that is able to also detect motifs located in domains. The fact that we do not find a pattern for unspecific cleavage sites (e.g. for the Clp proteases) shows that the method is also capable of separating random peptides from functional ones at the stage of motif derivation, should random peptides have been accepted by the SVM. Some motifs that were only detected in interactions between a peptide and domain in the same protein (intrachain interaction) could not be confirmed as true DMIs upon visual inspection and thus, we cannot assume to derive motifs accurately from intrachain data alone. This issue may improve if intrachain DMI data would be included in the training data, which is currently not the case as no reliable collection of intrachain peptide-mediated interactions is available.

It should also be noted that our final list of DMIs (Table 1 and Fig. 5) only includes those cases that could be confirmed in the interactome cross-validation, even though we know that some real cases, like the cytoskeleton-associated proteins domain (CAP_GLY) and many other known ELM motifs, are not significantly enriched in the current interactomes. This issue may improve with growth of protein interaction databases. Likewise, newly solved 3D structures may contain new DMIs, or raise information content for existing ones above the threshold required for application for our method. We cannot expect to recover the exact patterns described in ELM, which are manually curated and often exploit dedicated experiments to the relevance of a particular position or residue. Yet both our patterns and those from ELM score significantly in the datasets derived from 3D structures, and manual comparison shows that they are often similar. A potential problem is that the motifs we derive can only take sequences into account that occur in 3D structures, which may introduce a bias that would not be present in studies on all possible binding peptides. This might be addressed by applying methods such as iSPOT on the 3D structures identified here, combined with data from phage display scans [39]. On the other hand, we can include information on modified residues and particular spacing patterns in motif derivation, which are usually characteristic for a domain family and not just for individual instances. Recent studies have shown that the binding preferences of individual domains are probably too complex to be captured in regular expressions but that more complex models will be required [14], [33]. In addition to the physicochemical binding preferences of the domain, contextual factors will govern which interactions happen *in vivo* and which do not [18], [66]. The importance of the context may vary for different recognition domains and biological processes; for example, phosphorylation networks appear to heavily rely on contextual information [31], while a few (phosphorylation independent) domains have been shown to very specifically recognise the amino acid sequence of their binding partners [13], [30]. Again, more complex models will probably be required to integrate all this information that leads to the *in vivo* specificity of any given protein. Nevertheless, consensus motifs can be very helpful in studying commonalities among and differences between peptide-binding domains. A side effect of the large-scale derivation of motifs is that peptides are always given together with their binding domain in a way that can easily be transferred to other sorts of data, which is not always the case for manually curated DMIs. For example, by matching the pattern in one sequence and the binding domain in another may explain the mechanism behind some of the many protein-protein interactions which are currently being discovered in high-throughput interaction discovery experiments. This, in turn, may make them amenable to tinkering with this part of the network, either by designed peptides that might have fine-tuned affinity and specificity for the binding domain [63],[67] or by drug-like compounds that can interfere with the interaction [21], [34], [36], a long-standing dream of the pharmaceutical industry [35], [68], [69].

The information we exploit here to identify novel DMIs among the set of interactions of known 3D structure (i.e. well-defined structure upon binding), is of different nature than the one used by more traditional sequence and disorder-based methods applied by many other tools [24]–[26]. We think that our approach presents an extension to the currently available techniques, and should be regarded as complementary to them. Sequence-based discovery methods have access to much more data, especially with current high-throughput interaction and other functional association studies. The surface-searching method also accesses a larger pool of data, because more structures of individual domains are available than of DMI. Yet neither method can provide the level of detail we access here. The combination of these two kinds of data, with 3D structures to define motif interfaces and large sequence databases to establish evolutionary over-represented patterns, will certainly make a powerful predictor of linear motifs. On a limited scale, we already created such a hybrid method in the interactome cross-validation, yet more sophisticated implementations could include a wider variety of biological data and tackle the problem of capturing specificity, on the more abstract level of domain families as well as on the level of individual domains.

## Methods

### Control dataset

The control data is based on the Structural Classification of Proteins (SCOP) [44]. For each fold, we chose one representative structure and generated all possible peptides of 4–20 residues length. This dataset contains high overlaps among peptides from the same structure, which gives us a large variety of possible structural conformations of peptides to study. For the benchmark, we masked all peptides in regions covered by Pfam domains [46] and selected unmasked background peptides that had at least 4 contacts with a domain in a different protein chain as negative control cases. Thereby we ensured that negative cases had a chance to be identified as candidate peptides and would not be removed by our filters on peptides covered by domains and the minimum number of contacts.

### Structural parameters

The *Elongation* or “length” of a peptide is the distance between the 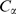 of the first and last residue of a peptide in Angstroms (Å). Because flexibility increases with peptide length in residues, short peptides have a small range of possible elongation values, while it varies more for long peptides. The *Linearity* is computed by constructing a line through the first and last 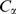 of each peptide, then calculating the distance of each 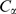 in the peptide to this line, and returning the maximum distance. A low value indicates a very flat or linear peptide. We used DSSP [45] for *secondary structure* assignments to peptides from the set of known DMI and from the SCOP background. The assignment is done on a single protein, after removing other chains in the structure but before extraction of the peptides, as DSSP does not always perform well on small fragments. Each peptide is assigned the DSSP class most frequently found among its residues. Note that because SVMs work on numerical data, including a more detailed description of the secondary structure of a peptide, such as the order of DSSP classifications, would require a much more complex model. We also used *accessibility* data computed by DSSP, and assigned the average accessibility of its residues to each peptide.

### Training of the Support Vector Machine (SVM)

We used the implementation “SVM-light” [70] and trained it on our data with a cost-factor of 10, meaning that errors in the classification of positive cases are 10 times worse than errors in the classification of negative cases, a trade-off between training error and margin of 0.1, and a linear kernel. These parameters were selected after searching the parameter space for different combinations of values for cost-factor, trade-off and kernel function, and testing recovery of known positive and negative (SCOP control set) cases. The estimation of classification errors also takes the fact that our set of positive cases is much smaller than the negative set into consideration.

### Interactions with neighbouring domains

To form a DMI, each peptide accepted by the SVM was also required to interact with a nearby domain, which may be part of the same protein or of another. Linear motifs usually form one connected interface with their binding domain, thus we excluded peptides in which there was a region of more than 4 residues that did not contact the domain, as well as those cases in which less than 60% of the peptide residues made contact with the domain.

### Interface size and ratio

We used NACCESS [71] to compute the interface between domain and peptide, and the “full interface” between the domain-containing protein and the peptide-containing protein (interchain DMIs) or between the domain and the rest of the protein (intrachain DMIs). In general, to accept a peptide-mediated interaction, the domain-peptide interface has to constitute at least 50% of the full interface. To accommodate for different stoichiometries in domain-peptide interactions (multiple domains binding one peptide), the threshold for the interface ratio was set to 0.5/*N*, where *N* is the number of domains involved in the interaction. If domains do not contribute roughly equally to an interaction or, more exactly, if any domain contributes less than *N/0.5*, they are removed in a filtering step. Since this changes the number of domains involved in the putative DMI and hence the minimum interface size, we repeated the filtering until each domain-peptide interface contributed appropriately for its stoichiometry and no domain was removed any more in the given step; in other words, we repeated the procedure until convergence. Note that, while all domain-peptide-interfaces have to contribute at least *0.5/N*, they do not necessarily contribute equally.

### Clustering by topology

We first aligned all sequences for a given domain to the corresponding Pfam HMM profile [46]. The aligned positions are used as a normalized numbering of the sequences, allowing easy comparison and mapping of corresponding positions. Next we computed the contacts for each domain-peptide interaction, and compared for each pair of domains how many of the corresponding domain positions contacted the peptide in both cases (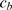). The resulting distance score is 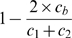, with 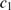 and 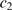 being the number of domain positions involved in contacts for the two respective domains. If the interface sizes are vastly different (one has more than double the contacts of the other), we set the score to 1, as these interfaces are considerably different despite possible overlaps (visual inspection). After computing the distance matrix for all DMIs involving the given domain, we clustered the interaction topologies by complete linkage hierarchical clustering [72] and cut the resulting tree at a distance of 1, which corresponds to no shared contacts (or artificially separated cases with large differences in interface size, cf. above).

### Computing peptide regions for overlapping peptides

Each continuous part of a protein that was covered by one or more peptides was designated as a peptide-containing region. These regions are non-overlapping by definition, and they represent parts of the protein that contain one or more peptides structurally similar to those found in known DMIs. The main motivation for this was that each pattern match in a given structure should only be counted once for “support” of that pattern, independent of how many accepted peptides include it (cf. Fig. 3).

### Clustering by sequence

For the sequence-based clustering, domain and peptide alignments are computed individually, and then the pairwise similarities are combined into one score, which is then used for clustering. For domains, all sequences of a given family are aligned to the Pfam HMM profile, and the sequence identity is computed from this alignment, yielding a pairwise domain sequence identity score 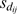 for each pair of domains. The corresponding peptide-containing regions are aligned using the Needleman-Wunsch algorithm [73], yielding a pairwise peptide sequence identity score 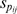 for each pair of peptides. The distance score is then computed as 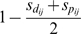 for each pair *ij*, where *i* and *j* are candidate domain-peptide interactions. Like for the topological clustering, we applied complete linkage hierarchical clustering [72]. Note that we use both domain and peptide sequences, because for the diversity of a DMI it makes a difference whether a given peptide is always bound by the same domain or by different domains. As a cut-off here we chose 0.1, which corresponds to 90% sequence identity. Thus, all resulting clusters have a combined sequence identity of 90% considering both domains and peptides.

### Motif derivation

We used SLiMFinder [25] to derive consensus motifs for the sets of peptide sequences bound in each topological cluster. We computed the amino acid frequencies from all sequences in the PDB, and disabled the ‘termini’ flag because beginnings and ends of our sequence fragments usually do not correspond to actual protein termini. “Unrelated protein clusters” (UPCs) were defined using the sequence-based clustering described above. Only topological clusters with 3 or more UPCs were searched for consensus motifs; the information content is too low in the other cases.

### Enrichment of DMIs in interactome networks

We created interactomes for human, fly, worm and yeast by integrating protein-protein interaction data from the databases MINT, IntAct and HPRD [74], [75], [76] that are supported by peer-reviewed publications. To ensure species specificity, we excluded hybrid interactions observed between proteins from different species, resulting in networks with 53,290 (human), 19,260 (fly), 5,566 (worm) and 60,721 (yeast) interactions, respectively. As described above, we only considered interactions that could not be explained by domain-domain interactions as observed in 3D structures [10], which reduces the interactomes to 43,882, 18,113, 5,234 and 58,426 edges, respectively. These interactomes involve 7,808 human, 6,610 fly, 3,111 worm and 5,266 yeast proteins, respectively.

To calculate motif enrichments in the interactome networks, we assigned Pfam domains, via HMM profiles, to all proteins in the respective interactomes, and tested for motif hits by regular expression pattern matching, only considering regions outside domains as described above. We then created a contingency table for each motif and species stating how many proteins contained at least one motif match, and how many interact with a protein containing the motif's binding domain. The enrichment factor was computed as 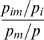, where 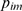 is the number of proteins that interact with another protein know to contain the binding domain and also have a motif match, 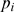 is the number of proteins that interact with another containing the binding domain, 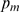 is the number of proteins with a motif match, and *p* is the total number of proteins in the interactome. The p-value was computed using Fisher's exact test on the contingency table, as implemented in R [77].

## Supporting Information

Figure S1 Linearity and Elongation of known motifs in comparison to a background sampling. (a) Linearity, (b) Elongation, (c) Linearity for 7-residue-peptides, split by DSSP-classification, (d) Elongation for 7-residue-peptides, split by DSSP-classification. The distribution of values varies greatly across and within classes of secondary structure.
(0.14 MB EPS)

Figure S2 (a) Interface size for known domain-peptide interactions. (b) Ratio of the interface between domain and peptide to the full protein-protein interface for the known DMI. Both are computed as described in the Methods section of the main manuscript.
(0.77 MB EPS)

Table S1 All DMI candidates derived from high-resolution 3D structures, specifying the binding domain, topology or interaction type ID, the consensus motif, enrichment and p-value in the interactome cross-validation, if applicable, and the ELM pattern and name, if available. For each interaction type, up to 3 top-ranked motifs are provided.
(0.05 MB XLS)
